# Soluble immune markers in the different phases of chronic hepatitis B virus infection

**DOI:** 10.1038/s41598-019-50729-5

**Published:** 2019-10-01

**Authors:** Steffen B. Wiegand, Bastian Beggel, Anika Wranke, Elmira Aliabadi, Jerzy Jaroszewicz, Cheng-Jian Xu, Yang Li, Michael P. Manns, Thomas Lengauer, Heiner Wedemeyer, Anke R. M. Kraft, Christine S. Falk, Markus Cornberg

**Affiliations:** 1 Department of Gastroenterology, Hepatology and Endocrinology, Hannover Medical School, Hannover, Germany; 2 German Centre for Infection Research (DZIF), partner-site, Hannover, Braunschweig Germany; 3 University of Applied Sciences, Kaiserslautern, Germany; 4 Department of Infectious Diseases and Hepatology, Medical University of Silesia, Katowice, Poland; 5 Department of Computational Biology and Applied Algorithmics, Max Planck Institute for Informatics, Saarland Informatics, Saarbrücken, Germany; 6 Institute of Transplant Immunology, Hannover Medical School, Hannover, Germany; 7 Department of Gastroenterology and Hepatology, University Hospital Essen, Essen, Germany; 8 Centre for individualized infection medicine (CIIM), Hannover, Germany; 9 Helmholtz Centre for Infection Research, Braunschweig, Germany

**Keywords:** Chemokines, Hepatitis B

## Abstract

Chronic hepatitis B virus (HBV) infection may follow four different consecutive phases, which are defined by virology as well as biochemical markers and differ in terms of prognosis and need for antiviral treatment. Currently, host responses reflected by immune markers are not considered in this definition. We aimed to study soluble immune markers and their distribution in different phases of chronic HBV infection. In this cross-sectional retrospective study, we investigated a panel of 14 soluble immune markers (SIM) including CXCL10 in 333 patients with chronic HBV infection. In a small cohort of HBeAg positive patients we analyzed SIM before and after HBeAg seroconversion and compared seroconverters to patients with unknown outcome. Significant differences were documented in the levels of several SIM between the four phases of chronic HBV infection. The most pronounced difference among all investigated SIM was observed for CXCL10 concentrations with highest levels in patients with hepatitis. TGF-β and IL-17 revealed different levels between HBeAg negative patients. HBeAg positive patients with HBeAg seroconversion presented higher amounts of IL-12 before seroconversion compared to HBeAg positive patients with unknown follow up. SIM such as CXCL10 but also IL-12, TGF-β and IL-17 may be useful markers to further characterize the phase of chronic HBV infection.

## Introduction

Hepatitis B virus (HBV) is a global health burden with almost 250 million chronically infected people worldwide^1^. Chronic hepatitis B can lead to liver cirrhosis and hepatocellular carcinoma (HCC)^2^. The clinical courses differ remarkably from patient to patient and not all will develop cirrhosis and/or HCC. Therefore, early identification of patients with high risk of developing endpoints like cirrhosis and HCC is necessary, in order to define who is in need of close monitoring and/or antiviral treatment^2^.

Four phases of the natural course of chronic HBV infection are characterized, which are based on serological and biochemical parameters^2^. The different phases of chronic HBV infection are not necessarily sequential. HBeAg-positive patients are usually young patients with very high HBV DNA titers. A differentiation between HBeAg-positive HBV infection (EPI) (formally called immune tolerance phase) and the HBeAg-positive hepatitis phase (EPH) is based on the occurrence of inflammatory signs such as elevated transaminases or histology-proven inflammation and/or clinical hepatitis^2^. After seroconversion to anti-HBe, HBeAg-negative patients with low HBV DNA level (<2,000 IU/mL) and no signs of liver inflammation are part of the HBeAg-negative infection (ENI) (formerly inactive carrier). HBeAg-negative patients with HBV DNA > 2,000 IU/ml and elevated ALT or liver inflammation due to immune escape mutations in the pre-core or basal core promoter are defined as having HBeAg-negative hepatitis (ENH).

Current clinical practice guidelines recommend antiviral treatment in patients with active hepatitis phases (EPH and ENH) but not in patients with HBeAg-positive or negative infection (EPI or ENI)^2^. However, a proportion of patients with HBeAg-negative chronic HBV infection and persistently normal ALT level have HBV DNA levels between 2,000 and 20,000 IU/ml.

These patients are sometimes defined as grey-zone patients and some data suggest that these patients have no significant liver disease and no increased risk for disease progression^3^. However, long-term follow-up is important to define the phase. Since quantitative HBV DNA and ALT levels may fluctuate in the natural course, the discrimination of patients with or without hepatitis is sometimes challenging^2^. In these cases, quantitative HBsAg as an additional serological parameter can be used to discern different phases of chronic HBV infection, e.g. ENI versus ENH^4,5^. However, additional markers are needed to improve our understanding of HBV pathogenesis. Due to the importance of immune responses in controlling the virus and finally resolving HBV infection, cytokines and chemokines might be a promising approach to further optimize the prediction of the natural course of chronic HBV infection. In patients infected with hepatitis C virus (HCV), several studies could show the importance of CXCL10 or also known as IP-10 (IFN-γ-inducible protein of 10 kD) in prediction of response in patients undergoing combination therapy of pegylated interferon α (PEG-IFN) and ribavirin^6^ or spontaneous clearance of acute HCV infection^7^. CXCL10 may also play an important role in HBV infection^8^, for example, CXCL10 is up-regulated in the liver during acute HBV infection in chimpanzees^9^. In HBV/HCV co-infected patients, dominance of HCV was associated with higher CXCL10 and lower quantitative HBsAg levels^10^. In HBV patients undergoing therapy with PEG-IFN or nucleos(t)ide analogues (NA), high baseline CXCL10 level were associated with HBsAg decline during therapy^11–14^.

In this study, we assessed 14 soluble immune markers (SIM) including CXCL10 in different phases of chronic HBV infection in a large European cohort to identify additional immune marker, which should help to differentiate the phases of chronic HBV infection.

## Material and Methods

### Patient population

Three hundred thirty-three European patients with persistent HBV infection, not undergoing antiviral therapy, were included in this cross-sectional study (Table 1).

**Table 1:** Baseline characteristics and factors univariately differentiating between patients in different phases of chronic HBV infection based on ANOVA (continuous values) and Chi-Square analysis (discrete values).

	EPI (n = 45) HBV-DNA ≥ 10^7^ IU/ml, ALT < ULN*	EPH (n = 89) HBV-DNA > 2,000 IU/ml, ALT ≥ 1.5ULN	ENI (n = 88) HBV-DNA < 2,000 IU/ml, ALT < ULN	ENH (n = 63) HBV-DNA ≥ 2,000 IU/ml, ALT ≥ 1.5ULN^#^	ENI-HR (n = 48)HBV-DNA 2,000–20,000 IU/ml, ALT < ULN	ANOVA p-value or χ^2^
Age, yrs (median, min.-max)	18 (1–49)	28 (7–68)	41 (12–69)	41 (22–67)	29 (14–63)	<0.001
Gender, n (M/F)	25/20	66/23	40/48	41/22	18/30	<0.001
HBV DNA log_10_ IU/mL (median, 10–90% CI)	8.07 (7.75, 9.25)	8.04 (5.23, 8.98)	2.29 (1.08, 3.11)	5.56 (3.95, 8.01)	3.83 (3.39, 4.22)	<0.001
HBsAg log_10_ IU/mL (median, 10–90% CI)	4.96 (4.34, 5.36)	4.31 (3.46, 5.16)	3.11 (0.87, 4.11)	3.91 (2.77, 4.39)	3.79 (3.05, 4.36)	<0.001
HBV Genotype, n:						0.103
A	2	23	14	14	5	
B	8	8	2	2	1	
C	2	5	1	3	0	
D	30	39	38	35	19	
E	0	1	0	1	0	
F	0	1	0	0	0	
not performed	3	12	33	8	23	
ALT (ULN) (median, 10–90% CI)	0.78 (0.53, 1.03*)	2.69 (1.59, 10.80)	0.65 (0.42, 0.92)	2.35 (1.39^#^, 11.69)	0.67 (0.43, 0.89)	<0.001
Bilirubin (ULN) (median, 10–90% CI)	0.41 (0.19, 0.82)	0.53 (0.24, 1.34)	0.47 (0.29, 0.82)	0.73 (0.29, 1.20)	0.53 (0.28, 1.19)	0.038
Platelets, 10^6^/µL (median, 10–90% CI)	242 (183, 335)	198 (127, 292)	220 (152, 298)	189 (103, 251)	224 (168, 286)	<0.001
cirrhosis n (%)	1 (2.2)	12, (13,4)	10 (11.3)	6 (9.5)	2 (4.2)	0.301

*In EPI group patients with ALT < 1.5ULN were accepted if no inflammation in liver biopsy was observed.

^#^In ENH group patients with ALT 1.0–1.5 ULN were accepted if significant inflammation (A1 > 2) in liver biopsy was observed.

Patients were recruited at the outpatient clinic of Hannover Medical School, Germany. One hundred thirty-four patients were HBeAg positive and 199 were HBeAg negative. They included 190 males and 143 females with a median age of 32 years (min. 1, max. 69 yrs.). None of the included individuals were co-infected with hepatitis δ virus (HDV), hepatitis C virus (HCV) or human immunodeficiency virus (HIV). Further exclusion criteria were end-stage liver insufficiency, alcoholism, autoimmune disorders, immunosuppressive treatment and malignancies. Patients were divided according to their phase of chronic HBV infection or hepatitis defined according to EASL^2^:

- HBeAg-positive chronic infection (EPI, n = 45)
- HBeAg-positive chronic hepatitis (EPH, n = 89),
- HBeAg-negative chronic infection (ENI, n = 88)
- HBeAg-negative chronic hepatitis (ENH, n = 63)
- HBeAg-negative chronic infection, high replicative (HBV DNA > 20,000, ALT normal) (ENI-HR, n = 48).

The high replicative status in HBeAg negative chronic infection (ENI-HR) was assessed, when DNA levels were between 2,000 and 20,000 IU/mL in at least two consecutive time-points with ALT levels less or equal the upper limit of norm (ULN).

For a 2^nd^ analysis a subpopulation of patients with chronic HBV infection (n = 39 out of 333) and patients with acute HBV (n = 10) infection or patients with suppressed HBV-DNA under nucleos(t)ide analogues (NA) therapy were included.

### Serum HBsAg quantification

Abbott ARCHITECT assay (Abbott Diagnostics, Abbott Park, IL) was used to quantify serum HBsAg levels.

The dynamic range of the test is 0.05–250 IU/mL. Samples were diluted 1:100 in horse serum and if HBsAg levels were >250 IU/mL, samples were retested at a dilution of 1:500 and 1:1,000, respectively. Samples with HBsAg levels <0.05 IU/mL were retested without prior dilution. Results are given in IU/mL.

### HBV-DNA measurement

Serum HBV-DNA was quantified by usage of COBAS AmpliPrep/COBAS TaqMan (Roche Diagnostics, Mannheim, Germany) and TaqMan Universal Master Mix (Applied Biosystems, Foster City, CA) with detection limits of 12 IU/mL and 50 IU/mL, respectively. HBV-DNA results are expressed in IU/mL.

### HBV genotyping

HBV genotyping was assessed in 254 patients. DNA was extracted from 200 µL of serum using High Pure Viral Nucleic Acid K15it (Roche Diagnostics, Mannheim, Germany) according to the manufacturer’s protocol. HBV genotype was determined by a modified RFLP PCR assay with amplified HBV-DNA by use of a nested PCR as previous described^15^. The sequences abstracted were matched with the National Center for Biotechnology Information GenBank and compared with described HBV-prototypes (Accession No. for: HBV-A Z72478; HBV-B D00329; HBV-C X01587; HBV-D V01460; HBV-E X75658; HBV-G AF160501). The HBV S sequence and related references sequences were aligned with CLC Main Workbench 6 (CLCBio, Aarhus, Denmark). Genetic distance was estimated also by use of CLC Main Workbench 6. Bootstrap resampling and reconstruction were carried out 1,000 times.

### Quantification of soluble immune mediators (cytokines and chemokines)

The concentrations of following SIM were measured in sera/plasma using the Luminex-based multiplex technology. In all patients CXCL10 (IP-10), IL-2, IL-4, IL-7, IL-10, IL-12p70 (IL-12), IL-16, IL-17, Interferon-γ, CCL4 (MIP-1β), TNF-α, TGF-β1, -2, -3 were measured and in the subpopulation additional concentrations were assessed for: IL-1β, IL-1R, IL-5, IL-6, IL-8, IL-9, IL-13, IL-15, Eotaxin, FGF-β2, G-CSF, GM-CSF, MCP-1, CCL3, PDGF-BB, RANTES, VEGF, IL-1α, IL-2Rα, IL-3, IL-12p40, IL-18, CTACK, GRO-α, HGF, IFN-α2, LIF, MCP-3, M-CSF, MIF, MIG, β-NGF, SCF, SCGF-β, SDF-1α, IL-2, TNF-β, TRAIL, sCDL40.

Bio-Plex assays (Bio-Rad, Hercules, USA) contain standard concentrations of each analyte and the calculation of respective standard curves allows for a precise definition of the concentrations of the protein of interest. The assay was performed according to the manufacturer’s protocol. In brief, lyophilized cytokine standard was resuspended in standard diluent. Serial dilution series were performed to generate standard curves for each cytokine, chemokine and growth factor of interest. The bead mixture, specific for cytokines, chemokines was incubated for 30 min at RT with 50 μL standard or serum samples that were diluted in sample diluent (1:2 dilution). Several washing steps were performed with 100 μL wash buffer/well, using the automated washer for magnetic beads (Bio-Rad). For the quantification of TGF-ß1-3, according to the manufacturer’s protocol, an acidification step was introduced by adding 5 µL HCl to the serum samples for 15 min in order to release TGF-ß from its binding proteins. After a normalization step with NaOH back to pH7, all samples were added to the bead mixture and processed with the same protocol like all other analytes. After addition of secondary biotinylated antibody mix for 30 min at RT and three more washing steps, SAPE was added for 10 min at RT (1:100 dilution).

After three final washing steps, beads were resuspended with 125 μL assay buffer, acquired and analyzed by the BioPlex Manager 6.0 software.

### *In-vitro* Peptide stimulation and intracellular staining (ICS)

To study the effect of IL-12 on T cells, thawed PBMCs from two EPI patients were stimulated for 10 days with 2.5 µg/mL HBV core overlapping peptides (OLPs) (Genotype D, Proimmune, UK) in presence of 10 ng/ml rhIL-12 (Miltenyi Biotech). 41 HBV core OLPs were divided into 2 pools; HBV core peptides #1 including 20 peptides and HBV core peptides #2 including 21 peptides. Cultures were supplemented with 5 U/ml rhIL-2 (Invitrogen Life Technologies) at days 4 and 8. Cells were restimulated at day 10 with the same HBV core OLPs and rhIL-12 for 6 hours. 2 µg/ml Brefeldin A (Sigma-Aldrich) was added after 1 hour of restimulation. For cell surface staining, PBMCs were stained with CD3 (Biolegend), CD14 (BD Bioscience), CD19 (BD Bioscience), CD4 (BD Bioscience) and CD8 (Biolegend). Dead cells were excluded using the Aqua cell stain kit (Life technologies). After fixation and permeabilization of PBMCs with Foxp3/Transcription Factor Staining Buffer Set (Ebioscience/Thermofisher), they were stained for IFN-γ (BD Bioscience) and TNF (Biolegend) for 30 min. Samples were acquired using BD LSR Fortessa (BD Biosciences) and data were analysed in FlowJo version 9.9.4 (Treestar).

### Statistical analyses

Statistical analyses were performed by using SPSS software version 21 (SPSS Inc., Chicago, IL). P-values < 0.05 were considered as statistically significant. If not otherwise noted, all values mentioned are the median and 10–90% confidence interval. Correlation was tested with the Spearman correlation test.

A chi-square test was calculated for the comparison of discrete variables. For univariate analyses, we applied the Mann-Whitney U test for continuous data. Multivariate analyses were performed with the Kruskal-Wallis ANOVA. Multivariate logistic regression analyses were performed by using the likelihood ratio test for backward selection.

To prevent bias from different types of samples (sera/plasma), median values were calculated at first. The quotient of median in plasma results divided by median in serum results was multiplied by each serum result for each cytokine. SIM concentrations were logarithmized (log10) in order to utilize parametric mathematics.

For analysis of cytokine patterns an outlier analysis was performed. For evaluation we used a 50-fold median threshold and a 1/50th median threshold in each group. All parameters, which were not located in this range, were deleted for the following analysis.

### Ethical approval

This retrospective study was conducted in accordance with the guidelines of the Declaration of Helsinki, the principles of Good Clinical Practice and according to standards of the local ethics committee. All patients provided informed consent for the analysis of parameters assessed in stored blood samples taken during routine diagnostics. Data were analyzed anonymously. The local ethical committee of Hannover Medical School approved the retrospective, anonymous retesting of patient samples and the anonymous analysis of patient data.

## Results

In this study 333 HBsAg positive patients were grouped in four consecutive phases of chronic HBV infection (EPI, EPH, ENI, ENH) and in the HBeAg-negative chronic infection with high replication (ENI-HR) (Table 1). HBeAg-positive patients (ENI and EPH) were significantly younger than patients with HBeAg-negative chronic HBV infection or hepatitis (EPI: 18.8 + 9.9, EPH: 32.4 + 16.0, ENI: 40.9 + 14.6, ENH: 40.8 + 11.2, p < 0.001, [years]). The proportion of females in ENI and ENI-HR was higher than in the EPH and ENH phases. HBV genotype distribution and prevalence of liver cirrhosis was similar between the different phases of chronic HBV-infection (Table 1).

Baseline characteristics of patients in the 2^nd^ subpopulation with concentrations of 52 SIM are shown in Table S1.

### Distribution of HBsAg levels

**Figure 1:**
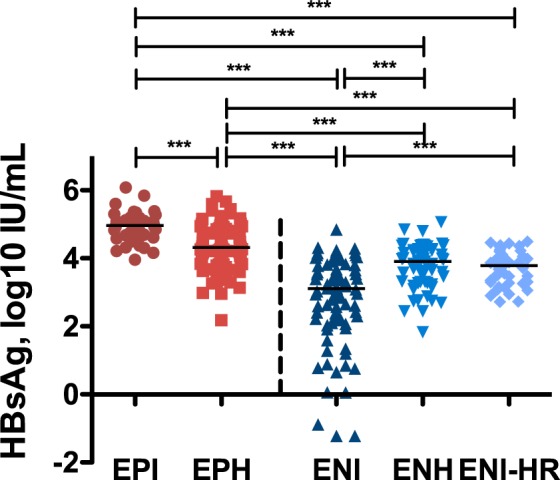
qHBsAg titers in chronic HBV. HBsAg titers in different phases of chronic HBV infection were quantified by using Abbott ARCHITECT assay (red: HBeAg positive, blue: HBeAg negative). Mann-Whitney U test was used for comparison of means.

Serum HBsAg levels differed between the different phases in chronic hepatitis B patients (Fig. 1). Levels were highest in EPI patients (4.96 log10 IU/mL). In the EPH group the median HBsAg titer was 4.31 log10 IU/mL, whereas in ENI the lowest median HBsAg result was observed (3.11 log10 IU/mL) (Fig. 1). Quantitative HBsAg (qHBsAg) data of the other two HBeAg negative phases (ENH and ENI-HR) compared with the results of EPH patients were more than 4.5- and 6.5-fold lower, respectively (3.91 log10 IU/mL and 3.79 log10 IU/mL) (Fig. 1). Interestingly, there was no significant difference in qHBsAg results between ENH and ENI-HR (p = 0.754).

### Distribution of cytokine and chemokine (soluble inflammatory marker) levels

**Figure 2:**
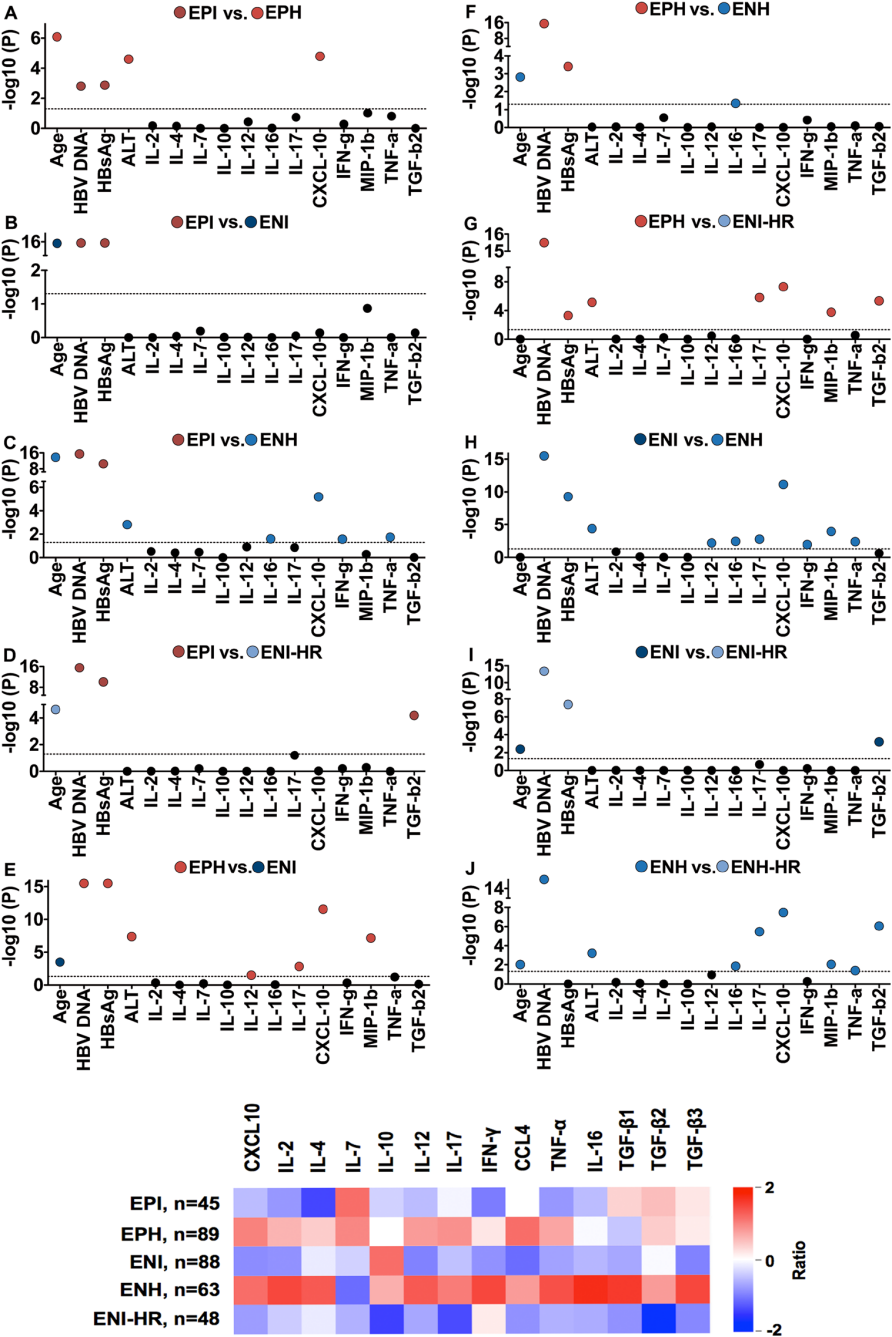
SIM in phases of chronic HBV. (**A**) Significance evaluation of different virological, biochemical and immunological markers between phases of chronic HBV infection. The broken line indicates the significance threshold. Nonsignificant differences are marked with black dots. Significant differences are marked with a colored dot. Colored dots according to group color connotes significantly higher values of the parameter in this group compared to the other group. (**B**) Heat map showing the expression pattern of 14 soluble immune markers normalized by setting mean = 1 and variance = 0. The elements are colored according to the value of each SIM for each phase.

Univariate ANOVA revealed several serological, biochemical and immunological parameters, which differed between the groups (Table 2; Fig. 2A).

**Table 2:** Evaluation of significant differences between different phases of chronic HBV patients by using Kruskal-Wallis ANOVA *(A* = *EPI vs. EPH; B* = *EPI vs. ENI; C* = *EPI vs. ENH; D* = *EPH vs. ENI-HR; E* = *EPH vs. ENI; F* = *EPH vs. ENH; G* = *EPH vs. ENI-HR; H* = *ENI vs. ENH; I* = *ENI vs. ENI-HR; J* = *ENH vs. ENI-HR)*.

Analyte	P value	Q Value	Group
HBV DNA	5.42 × 10^−120^	4.35 × 10^−119^	ABCDEFGHIJ
HBsAg	1.47 × 10^−37^	5.89 × 10^−36^	ABCDEFGHI
CXCL10	5.80 × 10^−18^	1.16 × 10^−17^	ACEGHJ
Age	1.13 × 10^−17^	1.81 × 10^−17^	ABCDEFIJ
ALT	4.23 × 10^−11^	3.15 × 10^−11^	ACEGHJ
CCL4	1.18 × 10^−8^	6.31 × 10^−9^	EGHJ
IL-17	3.92 × 10^−8^	1.97 × 10^−8^	EGHJ
TGF-β2	3.93 × 10^−7^	1.86 × 10^−7^	DGIJ
TNF-α	7.85 × 10^−4^	2.86 × 10^−4^	CHJ
IL-12p70	2.58 × 10^−3^	8.12 × 10^−4^	EH
IL-16	2.83 × 10^−3^	8.74 × 10^−4^	CFHJ
IFN-γ	9.99 × 10^−3^	2.82 × 10^−3^	CH

For a better visualization a heat map showing expression patterns of SIM were created (Fig. 2B and Supplementary Fig. S1).

**Figure 3:**
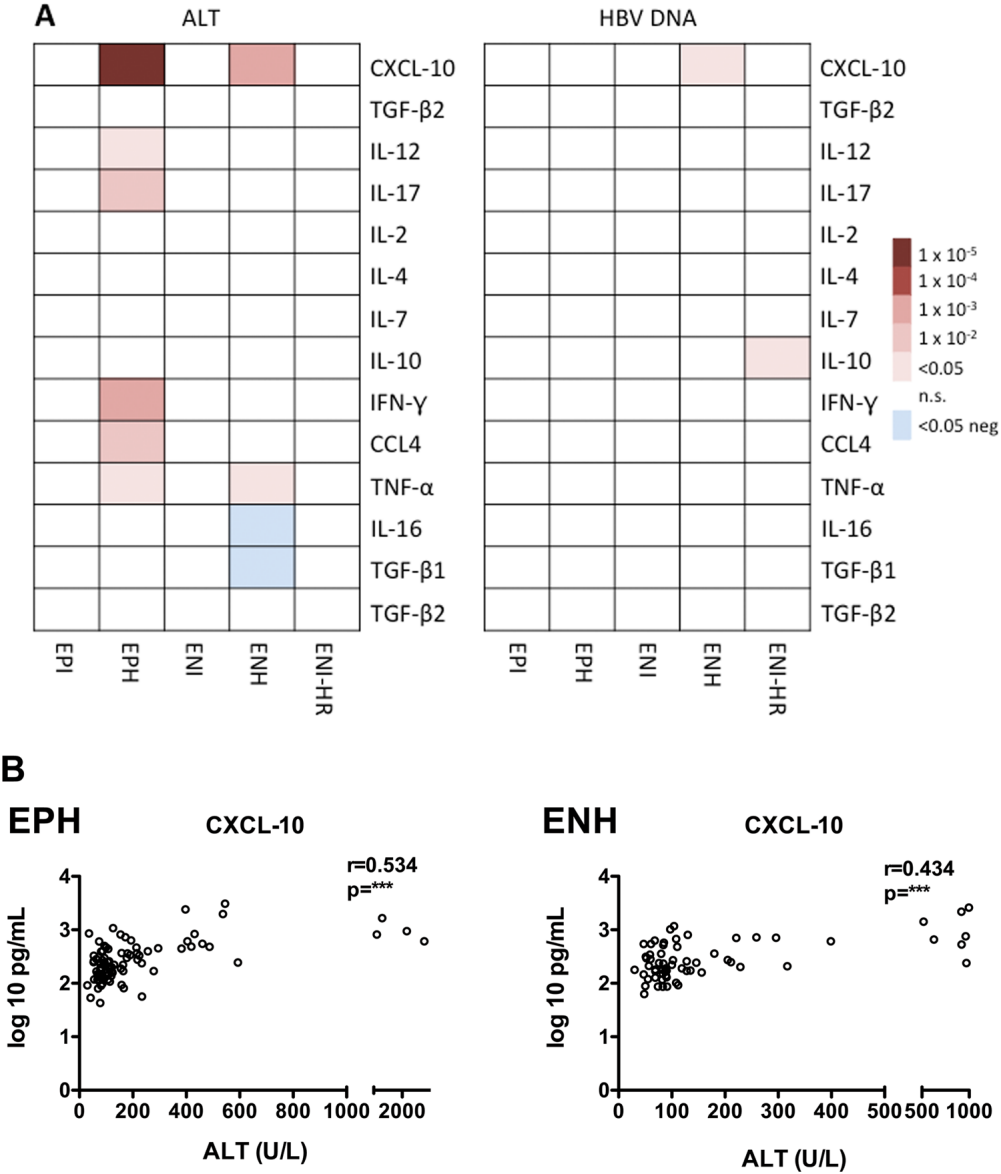
Correlation of SIM and ALT or HBV DNA. (**A**) Spearman rank correlation coefficients between SIM levels (rows) and ALT (left panel) or HBV DNA (right panel). Red indicates a significant positive correlation, whereas blue indicates a significant negative correlation. White color indicates non-significant (n.s.). (**B**) Spearman rank correlation between CXCL-10 and ALT in EPH (left) and ENH patients (right).

We have also tested the correlation between 14 cytokine levels and ALT and HBV DNA levels. We identified significant correlation between cytokine levels and ALT, especially in EPH and ENH, but weak correlation between expression levels and HBV DNA levels (Fig. 3A). CXCL10 concentrations showed the strongest correlation to ALT titers in EPH (r = 0.53, p < 0.001) and ENH patients (r = 0.43, p < 0.001) (Fig. 3B).

In line with this data, CXCL10 showed the strongest signal among all SIM considering differentiation of HBV phases.

**Figure 4:**
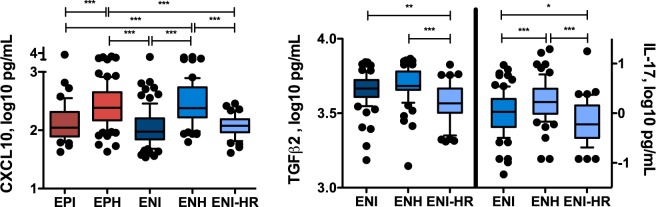
CXCL10, TGF-β2 and IL-17 titers in different phases of chronic HBV infection. (**A**) CXCL10 levels in HBeAg-positive and HBeAg-negative phases of chronic HBV infection (**B**) TGF-β2 and IL-17 levels in HBeAg-negative phases of chronic HBV infection. Mann-Whitney U test was used for comparison of means.

The highest CXCL10 concentrations were observed in the EPH and ENH patients with a median of 2.39 ± 0.36 log_10_pg/mL and 2.38 ± 0.36 log_10_pg/mL, respectively. Significantly lower CXCL10 concentrations were documented in patients without hepatitis in the EPI- (2.04 ± 0.36 log_10_pg/mL; p < 0.001), ENI- (1.98 ± 0.32 log_10_pg/mL; p < 0.001) or ENI-HR-phase (2.08 ± p0.19 log_10_pg/mL; p < 0.001) (Fig. 4A).

Among difficult-to-distinguish HBeAg negative individuals (ENI, ENH, ENI-HR) CXCL10, CCL4 (MIP-1β), IL-17, TGF-β1, -β2 and -β3, TNF-α, IL-12 (IL-12p70) and IL-16 differed significantly (Table 3) between these groups. CXCL10 concentrations were able to dissect ENH from ENI-HR and ENH from ENI, whereas IL-12 helped to distinguish ENI and ENH. ENI patients had lower IL-12 and CCL4 levels (−0.73 ± 0.84 log_10_pg/mL and 1.18 ± 0.28 log_10_pg/mL) compared to ENH patients (−0.19 ± 0.90 log_10_pg/mL and 1.38 ± 0.35 log_10_pg/mL, Mann-Whitney U test; p < 0.001, respectively).

**Table 3:** Significant differences of SIM concentrations in HBeAg negative phases of chronic HBV; Kruskal-Wallis ANOVA.

	ENI (n = 88)	ENH (n = 63)	HRC (n = 48)	ANOVA p-value
CXCL10*	1.98 (1.68, 2.40)	2.38 (2.02, 2.87)	2.08 (1.82, 2.32)	<0.001
CCL4*	1.18 (0.94, 1.55)	1.38 (1.08, 1.93)	1.22 (0.97, 1.50)	<0.001
IL-17*	0.03 (−0.49, 0.48)	0.23 (−0.15, 0.72)	−0.22 (−0.63, 0.34)	<0.001
TGF-β1*	4.33 (4.01, 4.74)	4.46 (4.20, 5.08)	4.33 (4.03, 4.59)	0.002
TGF-β2*	3.67 (3.55, 3.77)	3.68 (3.60, 3.82)	3.57 (3.37, 3.72)	<0.001
TGF-β3*	3.11 (2.77, 3.41)	3.14 (2.94, 3.49)	3.12 (2.59, 3.29)	0.007
TNF-α*	0.02 (−0.36, 0.60)	0.34 (−0.27, 1.64)	0.00 (−0.31, 0.76)	<0.001
IL-12*	−0.73 (−2.00, 0.23)	−0.19 (−1.91, 0.84)	−0.52 (−2.00, 0.24)	0.002
IL-16*	1.67 (1.32, 2.28)	1.88 (1.32, 2.87)	1.71 (1.26, 2.13)	0.001

*log_10_ pg/mL (median, 10–90% CI).

Interestingly, only TGF-β2 and IL-17 were useful to differentiate between ENI and ENI-HR.

In ENI individuals, median concentrations of TGF-β2 were 3.67 ± 0.14 log_10_pg/mL and thus higher than in patients classified to ENI-HR (3.57 ± R0.13 log_10_pg/mL; Mann-Whitney U test; p = 0.001). IL-17 levels were 0.03 ± 0.43 log_10_pg/mL in ENI patients and −0.22 ± 0.42 log_10_pg/mL in ENI-HR patients; Mann-Whitney U test; (p = 0.040) (Fig. 4B). Performing a multivariate analysis only TGF-β remained an independent parameter for the differentiation of ENI- and ENI-HR patients (p < 0.001).

For an easier understanding, we restricted further analyzes to TGF-β2 when referring to TGF-β, based on the observed correlation between TGF-β1 and TGF-β2 (r = 0.79; p < 0.001) and between TGF-β3 and TGF-β2 (r = 0.83; p < 0.001) (Figs S2a and S2b).

In a 2^nd^ smaller cohort of chronic HBV patients with available data for 52 SIM, CXCL10 and TGF-β data were confirmed. In addition, sCD40L, PDGF-BB and RANTES differed between various phases of chronic HBV infection (Table S2).

SIM concentration patterns of patients with four phases of chronic HBV infection (EPI-HR not included) and SIM concentrations of acute hepatitis B patients, patients with HBV-DNA suppression and healthy individuals are visualized in Fig. S3. Of note, CXCL10 values were high in patients with acute hepatitis B and low in healthy controls and NA treated patients with HBV suppression.

### Cytokines in patients with HBeAg seroconversion

For 10 HBeAg positive patients with no history of antiviral therapy (n = 5 EPH and n = 5 EPI) a second time point after seroconversion to anti-HBeAg was available (Table S3). The mean range between both time points was 4 years and 8 months (range 12 months–84 months).

Comparing only those HBeAg seroconverters, who were considered as part of the EPI population before seroconversion with the other EPI patients with unknown outcome, higher IL-12 levels (−0.13 ± 0.54 log_10_pg/mL vs. −0.86 ± 1.05 log_10_pg/mL; p = 0.029) were documented (Fig. S4A). In addition, the seroconverters were younger than the rest of the EPI patients (6 ± 4 years vs. 20 ± 10 years; p = 0.008). After HBeAg seroconversion the values for TNF-α and ALT were lower than before in those patients (−0.31 ± 0.27 log_10_pg/mL vs. 0.34 ± 0.38 log_10_pg/mL; p = 0.005 and 0.62 ± 0.13 ULN vs. 0.98 ± 0.19 ULN; p = 0.044).

Analyzes of HBeAg seroconverters formerly grouped to EPH phase showed no difference in cytokine values, HBsAg titers or levels of transaminases compared to patients in the EPH phase with no documented HBeAg seroconversion. When comparing HBeAg seroconverters classified as EPH patients before and after seroconversion, only IL-17 levels (0.47 ± 0.19 log_10_pg/mL vs. −0.28 ± 0.40 log_10_pg/mL; p = 0.032), showed significant differences.

### Effect of IL-12 on T cell responses

To confirm the previously published data that IL-12 has an impact of HBV-specific T cell responses^16^, we have investigated the *in-vitro* effect of IL-12 on HBV core peptide stimulated PBMCs isolated from two young HBeAg positive patients with high HBsAg levels. After 10 days *in-vitro* HBV core peptide stimulation, CD4^+^, as well as CD8^+^ T cells, showed higher IFN-γ/TNF production assessed by an intracellular cytokine assay after IL-12 plus HBV core peptide re-stimulation compared with peptide stimulation only (Fig. S4B).

## Discussion

The differentiation of the phases of chronic HBV infection is important to individualize the management of the patients. However, a single determination of HBV DNA, as well as disease activity markers, does not allow an immediate classification to one of the phases. Quantitative HBsAg (qHBsAg) titers may help to improve the differentiation between phases in HBV infection^4,5,17^. Our results confirm data from previous studies that HBsAg levels differ between the phases of chronic HBV infection^18,19^. However, qHBsAg could not distinguish patients with ENH and patients with high replicative HBeAg negative infection (ENI-HR). Importantly, our study showed that soluble immune mediators could be a valuable additional tool to better characterize patients with chronic HBV infection including those with HBeAg negative infection.

Among 14 tested SIM, CXCL10 was the most significant SIM that helped to distinguish the different phases of chronic HBV infection. This was confirmed in a second smaller cohort. CXCL10 is known to be a mainly pro-inflammatory chemokine which has, among other functions, an impact in pathogenesis of chronic inflammatory in miscellaneous infectious diseases. CXCL10 may play a role in regulating activity for cell proliferation and induction of apoptosis^20^. This is an explanation why CXCL10 concentrations are highest in phases of active hepatitis and in patients with acute hepatitis B and, thus, may have an impact as an immunological imprint in the prognosis of the disease. High CXCL10 levels may suggest stronger immune activation, which can lead to disease progression^21^.

However, this may sometimes even be beneficial i.e. if these patients receive an additional treatment.

Several studies could show that higher CXCL10 levels were associated with HBsAg decline and HBeAg loss in response to pegylated Interferon alfa therapy and in patients undergoing antiviral therapy with nucleos(t)ide analogues^11–14,22^. In HCV/HBV co-infected patients higher CXCL10 levels in HCV dominant patients were associated with lower HBsAg concentrations compared with HBV dominant patients or HBV mono-infected patients^10^. If HBV is controlled by immune responses, i.e. in inactive carriers (ENI), or in patients on NA therapy, CXCL10 levels are low. In fact, in that case, lower CXCL10 levels may indicate that patients may clear HBsAg later on^23^. Thus, it is important to interpret the value of CXCL10 in the context of the different phases of HBV infection.

In the few patients analyzed longitudinally, CXCL10 was not significantly higher in patients who developed spontaneous HBeAg seroconversion compared with all HBeAg positive patients. This may suggest that CXCL10 alone is not sufficient in this situation and as discussed above, additional antiviral therapy may be important. But interestingly, patients in the EPI phase who could experience HBeAg seroconversion had significantly higher levels of IL-12 than patients in the EPI phase with unknown follow-up. The number of patients tested is small, but IL-12 was described to have an impact in restoring Th1 T cell functions. Exhausted CD8^+^ T cells, which are present in chronic HBV infection and in part responsible for the persistence of HBV could gain their functionality back by induction of IL-12^16^. IL-12 is known to improve cytotoxicity, polyfunctionality and multispecificity of HBV-specific T cells. We could confirm the results shown by Schurich *et al*.^16^ that IL-12 could enhance the *in-vitro* HBV-specific T cell responses in two young HBeAg positive EPI patients. Thus, concepts to improve T cell responses including IL-12 induction may be considered for future immune therapies for chronic hepatitis B.

Importantly, patients with subsequent seroconversion were younger compared with those patients with unknown outcome. The higher IL-12 level in these young serooconverters suggests the presence of immune responses and is in line with recent data challenging the concept of immune tolerance in young patients with HBeAg positive infection^24^.

Another interesting result was the difference in transforming growth factor-beta (TGF-β) and IL-17 among HBeAg negative patients. ENI-HR patients had the lowest levels of TGF-β and IL-17. It has been shown that TGF-β is involved in the development of Th17 cells, which are the source of IL-17^25^. This is in line with our results that TGF-β correlates with IL-17. Under the induction of TGF-β CD4^+^ T cells may become regulatory T cells or Th17 cells, depending on other cytokines^26^. Th17 cells may contribute to HBV suppression but also inflammation. ENH patients show the highest level of TGF-β and IL-17 but due to other immune escape mechanisms this may not lead to HBV control. In this case Th17 T cells may contribute to immune activation and aggravation of chronic hepatitis B^27,28^. The Th17/IL-17 axis was described as fuel and flame of a sustained proinflammatory and profibrotic environment^29^.

This current study is one of the first larger cross-sectional studies with more than 300 patients presenting insights in different cytokine and chemokine levels in the different phases of chronic HBV infection. Limitations of this study were its retrospective design and in general a lack of follow-up data including clinical endpoints. The impact of the results was limited because this cross-sectional study provided no solid evidence to explain how the SIM influence disease progression and vice versa or if viral factors such as precore or BCP mutations influence the results. Prospective longitudinal studies following patients that switch the phases of chronic HBV infection are warranted to address this important question. In addition, the study in more than 300 patients was limited to only a small subset of SIM and we could not define thresholds for single SIM that could be used for clinical practice. Non-specific reasons for different SIM level cannot be excluded.

However, the results confirm that CXCL10 may have an important role in chronic HBV infection. In addition, IL-12 may be an interesting marker to predict HBeAg seroconversion in young patients with HBeAg positive infection and TGF-β/IL-17 could be helpful to further characterize HBeAg negative patients. Additional studies should investigate the role of immune marker in larger cohorts of patients with HBV infection.

## Supplementary information

Supplementary Information

## Data Availability

The datasets generated during and/or analysed during the current study are available from the corresponding author on reasonable request. All data generated or analysed during this study are included in this published article (and its Supplementary Information files).
