# Supplementary material for: Soluble immune markers in the different phases of chronic hepatitis B virus infection
Source: Sci Rep. 2019 Oct 1;9:14118. doi: 10.1038/s41598-019-50729-5 (PMC6773856; doi:10.1038/s41598-019-50729-5)
Supplement: Supplementary file 1 — Supplementary Information [file 41598_2019_50729_MOESM1_ESM.pdf]

Soluble immune markers in the different phases of chronic hepatitis B virus infection

Steffen B. Wiegand [1,2], Bastian Beggel [3], Anika Wranke [1,2], Elmira Aliabadi [1,2], Jerzy Jaroszewicz [4], Cheng-Jian Xu [1,8], Yang Li [8,9], Michael P. Manns [1,2,6], Thomas Lengauer [2,5], Heiner Wedemeyer [1,2,7], Anke R.M. Kraft [1,2], Christine S. Falk, [2,6] & *Markus Cornberg [1,2,8,9]

	EPI (n=9) HBV-DNA≥10[7]IU/ml, ALT<ULN*	EPH (n=10) HBV-DNA>2,000IU/ml, ALT≥1.5ULN	ENI (n=10) HBV-DNA<2,000IU/ml, ALT<ULN	ENH (n=10) HBV-DNA≥2,000IU/ml, ALT≥1.5ULN[#]	acute HBV patients (n=10)	HBV patients under NUC therapy (n=10)	healthy (n=10)	ANOVA p-value or χ^2
**Age, yrs (median, min.-max)**	10 (4-19)	22 (7-67)	43 (28-62)	42 (26-53)	39 (19-60)	44 (28-57)	43 (21-69)	<0.001
**Gender, n (M/F)**	7/2	6/4	6/4	7/3	6/4	6/4	6/4	0.975
**HBV DNA log_{10} IU/mL (median, 10-90% CI)**	8.35 (7.74-8.59)	8.16 (7.02-8.84)	2.20 (0.70-3.09)	6.29 (4.41-8.81)	3.76 (2.24-4.83)	0 (0-0)		<0.001
**HBsAg log_{10} IU/mL (median, 10-90% CI)**	4.96 (4.55-5.38)	4.64 (4.10-5.08)	2.75 (0.71-3.59)	3.78 (2.85-4.91)	3.62 (2.72-4.67)	3.42 (2.53-4.03)		<0.001
**HBV Genotype, n:**								0.283
**A**	0	1	1	2	1	3		
**B**	1	0	0	0	0	0		
**C**	0	0	0	2	0	0		
**D**	8	6	6	4	1	5		
**E**	0	0	0	0	0	0		
**F**	0	0	0	0	0	0		
**not performed**	0	3	3	2	8	2		
**ALT (ULN) (median, 10-90% CI)**	0.98 (0.60-1.00)	2.62 (2.05-9.79)	0.70 (0.50-0.90)	11.62 (1.82-22.19,)	32.8 (6.56-73.49)	0.8 (0.5-0.96)		<0.001

Supplementary Table 1 Baseline characteristics and factors univariately differentiating between patients in different phases of HBV infection based on ANOVA (continuous values) and Chi-Square analysis (discrete values).

Analyte	P value	Q Value	Group
**HBV DNA**	2.72×10^{-14}	1.37×10^{-13}	BCEFH
**HBsAg**	5.07×10^{-07}	1.27×10^{-5}	BCEH
**CXCL10**	2.83×10^{-3}	2.02×10^{-2}	ACEH
**TGF-β2**	2.26×10^{-3}	1.62×10^{-2}	BCEH
**TGF-β3**	1.52×10^{-3}	1.10×10^{-2}	BCEH
**TGF-β1**	1.38×10^{-3}	1.03×10^{-2}	BCE
**sCD40L**	1.19×10^{-2}	4.98×10^{-2}	BCE
**PDGF-BB**	1.44×10^{-2}	9.47×10^{-2}	CE
**RANTES**	4.57×10^{-2}	2.50×10^{-1}	B

Supplementary Table S2 Evaluation of significant differences of virologic paramateres and SIM concentrations between different phases of chronic HBV patients by using Kruskal-Wallis ANOVA

(A= EPI vs. EPH; B= EPI vs. ENI; C= EPI vs. ENH; E= EPH vs. ENI; F= EPH vs. ENH; H= ENI vs. ENH). Group D, G, I and J are missing, as ENI-HR was not included.

	EPI before seroconversion (n=5)	after seroconversion (n=5)	EPH before seroconversion (n=5)	after seroconversion (n=5)
**Age, yrs (median, min.-max)**	6 (4-13)	12 (9-20)	30 (14-67)	37 (17-69)
**HBsAg log_{10} IU/mL (median, 10-90% CI)**	4.96 (4.32-5.84)	4.17 (3.76-5.35)	4.11 (3.66-4.64)	3.87 (3.32-4.06)
**HBV DNA log_{10} IU/mL (median, 10-90% CI)**	8.45 (7.80-9.05)	3.16 (1.08-9.47)	7.14 (6.10-8.81)	4.01 (3.30-5.92)
**ALT (ULN) (median, 10-90% CI)**	0.98 (0.62-1.00)	0.62 (0.44-0.78)	5.06 (1.88-11.91)	1.00 (0.38-2.85)
**IL-2***	-0.68 (-1.10-0.12)	-1.10 (-1.10-0.18)	-0.47 (-1.10- -0.33)	-1.10 (-1.10-0.24)
**IL-4***	-0.51 (-0.51- -0.51)	-0.51 (-0.51- -0.51)	-0.51 (-0.51- -0.51)	-0.51 (-0.51- -0.51)
**IL-7***	-0.77 (-2.60- -0.67)	-2.00 (-2.60- -2.00)	-0.99 (-2.60- --0.64)	-2.00 (-2.60- -0.87)
**IL-10***	-0.89 (-0.89-0.27)	-0.89 (-0.89- -0.89)	-0.89 (-0.89- -0.76)	-0.89 (-0.89-0.37)
**IL-12p70***	-0.13 (-0.73-0.74)	-1.40 (-2.13-1.22)	0.29 (-0.73-0.79)	0.14 (-2.13-0.47)
**IL-16***	1.62 (1.33-2.04)	1.51 (1.33-2.06)	2.00 (1.56-2.55)	2.09 (1.69-2.30)
**IL-17***	0.32 (0.07-0.55)	0.16 (-0.59-0.45)	0.47 (0.10-0.60)	-0.28 (-0.59-0.34)
**CXCL10***	2.11 (1.85-2.40)	2.06 (1.76-2.83)	2.86 (2.17-3.29)	2.23 (1.71-3.01)
**IFN-γ***	-0.08 (-0.08-1.04)	-0.08 (-0.08- -0.08)	1.04 (-0.08-1.52)	-0.08 (-0.08-0.72)
**CCL4***	1.50 (1.19-1.74)	1.33 (1.07-1.82)	1.54 (1.09-1.79)	1.34 (1.24-1.54)
**TNF-α***	0.34 (0.05-1.07)	-0.31 (-0.72)	0.41 (0.05-1.77)	0.34 (-0.12-0.83)
**TGF-β2***	3.69 (3.50-3.73)	3.70 (3.67-3.79)	3.66 (3.61-3.68)	3.55 (3.28-3.76)

*log_{10} pg/mL (median, 10-90% CI)

Supplementary Table S3. Virological, biochemical and immunological characteristics of HBeAg positive patients before and after HBeAg seroconversion

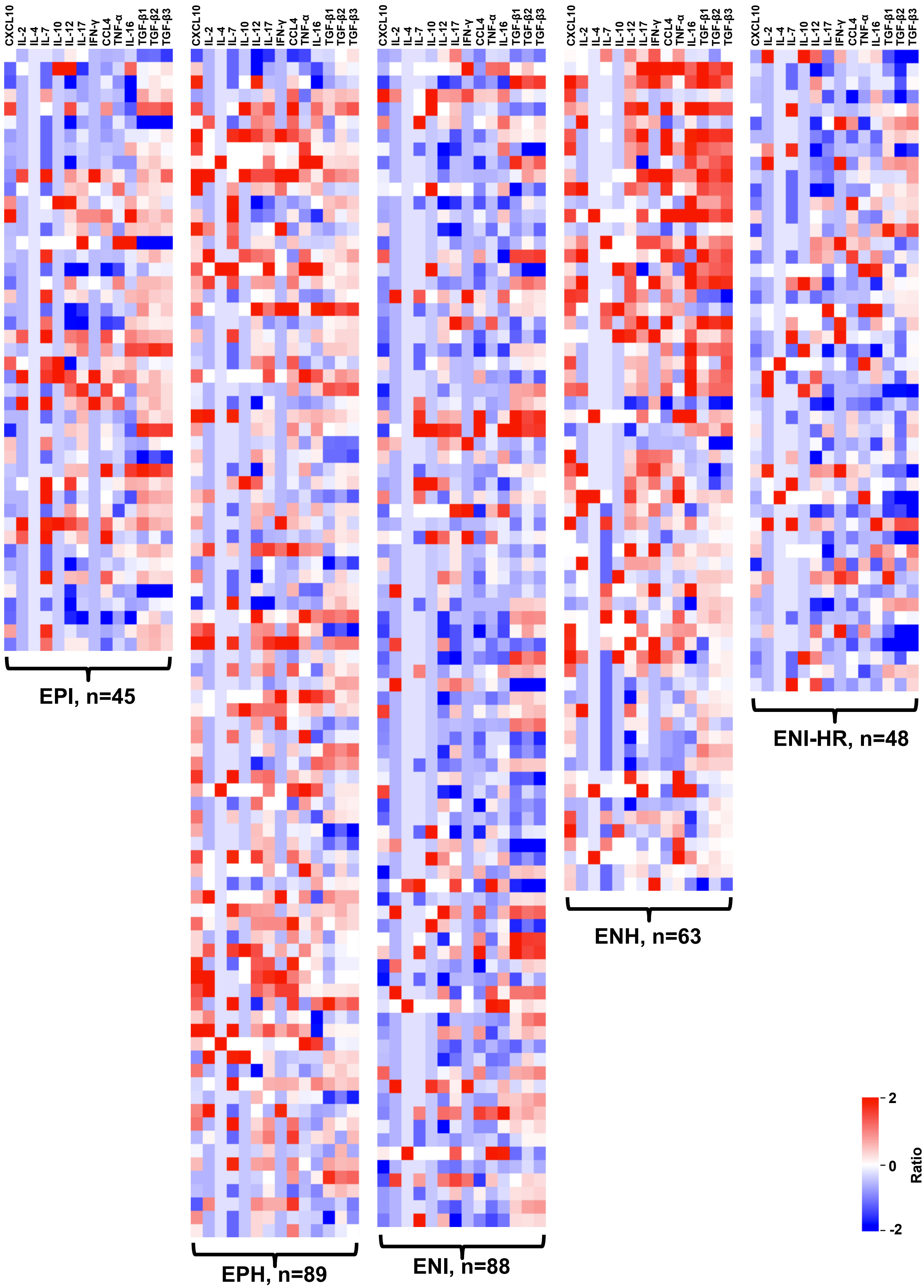
CXCL10
IL-2
IL-4
IL-7
IL-10
IL-12
IL-17
IFN-γ
CCL4
TNF-α
IL-16
TGF-β1
TGF-β2
TGF-β3
EPI, n=45
EPH, n=89
ENI, n=88
ENH, n=63
ENI-HR, n=48
2
0
-2
Ratio

Figure S1

Figure S1 Heat map distribution of 14 SIMs. Heat map showing the expression pattern of 14 soluble immune markers normalized by setting mean=1 and variance=0. The elements are colored according to the value of each SIM for each sample.

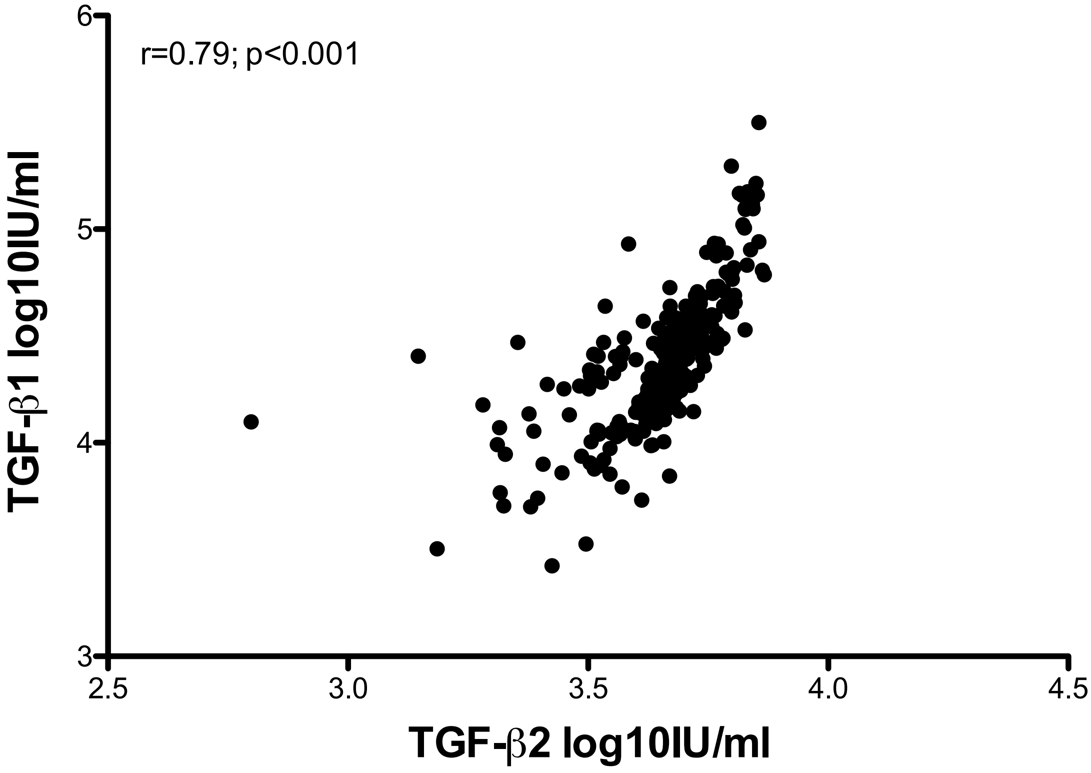

Figure S2a

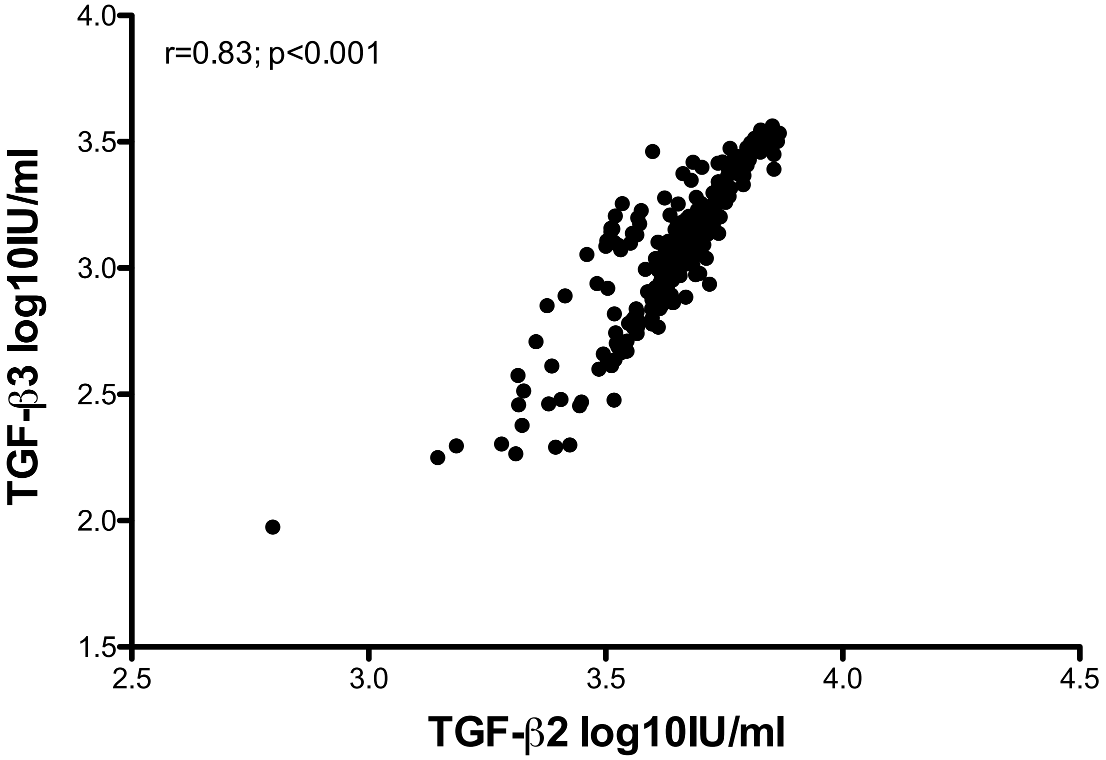

Figure S2b

Figure S2. Correlation between different TGF-β subtypes in chronic HBV patients. A) Correlation between TGF-β1 and TGF-β2. B) Correlation between TGF-β2 and TGF-β3

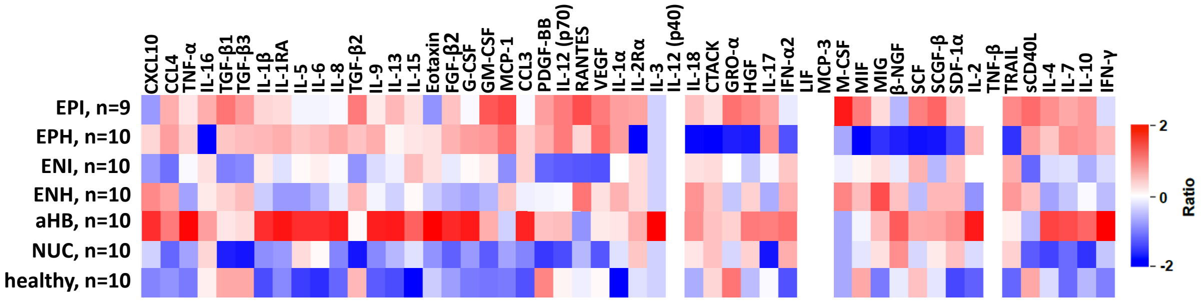

Figure S3 Heat map distribution of 52 SIMs. Heat map showing the expression pattern of 52 soluble immune markers normalized by setting mean=1 and variance=0. The elements are colored according to the value of each SIM for each sample.

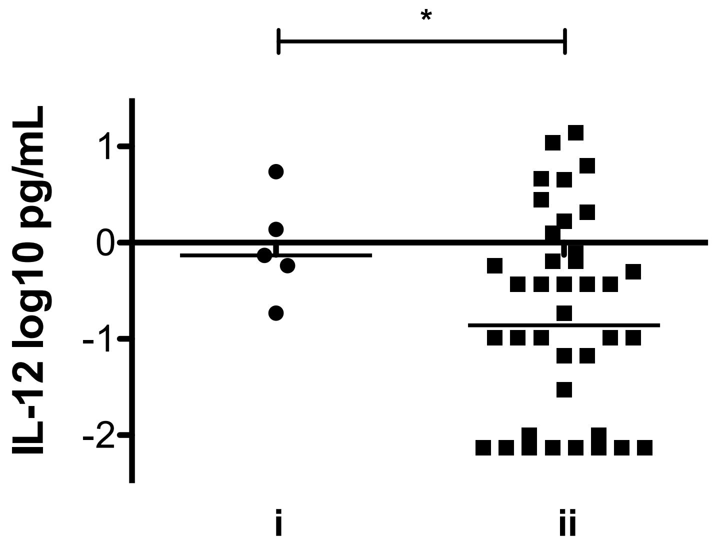

Figure S4a

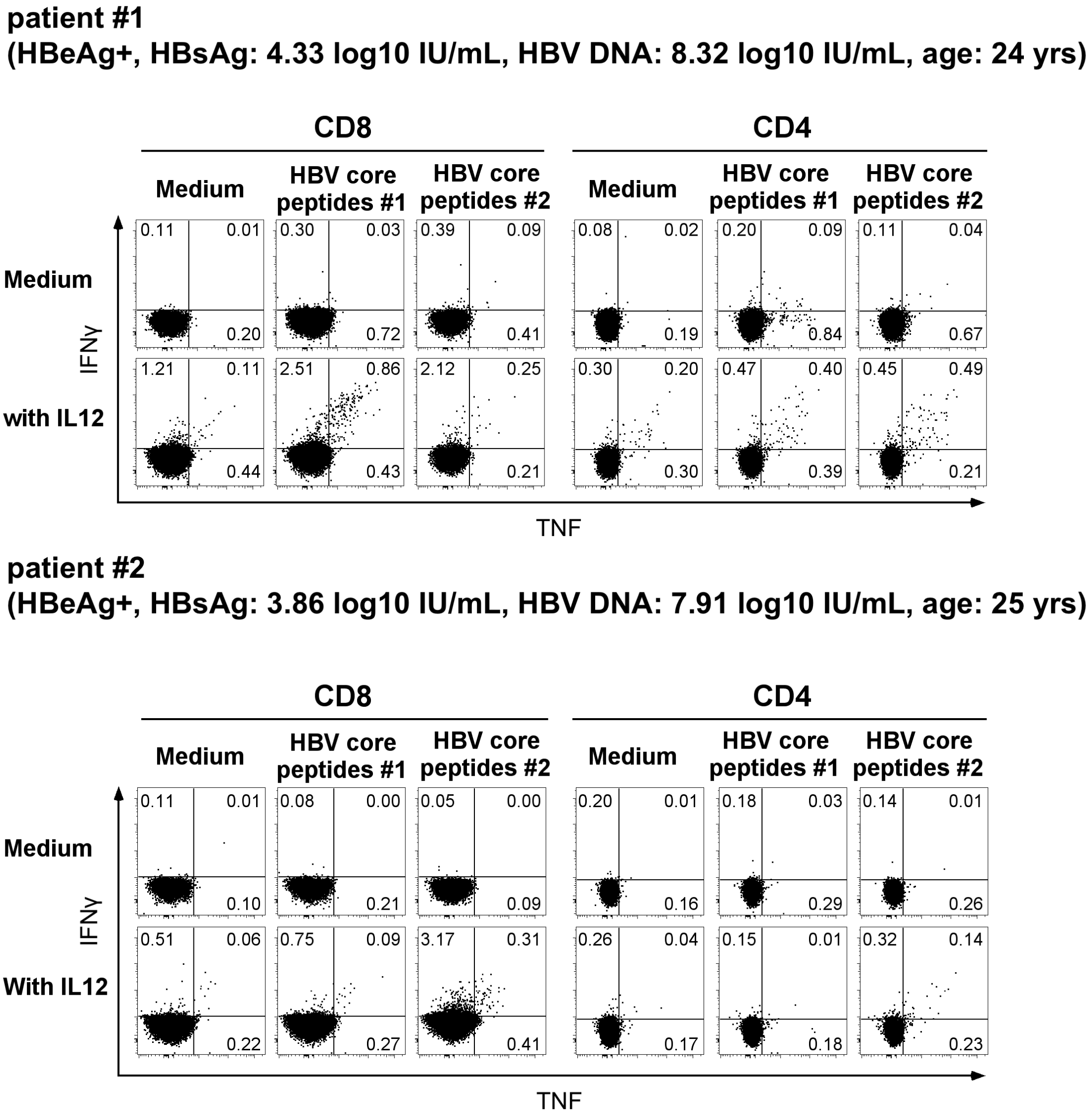

Figure S4b

Figure S4. IL-12 in EPI patients. A) IL-12p70 level comparison between HBeAg-positive patients who achieved HBeAg seroconversion (i) and patients with unknown follow-up (ii). Mann-Whitney U test was used for comparison of means B) Effect of IL-12 on HBV core-specific T cell responses. Representative FACS plots of $CD4^+$ and $CD8^+$ T cell responses in two EPI patients. PBMCs were stimulated with HBV core OLPs in the presence of rhIL-12. Lymphocytes were gated on singlets, live, $CD14^-$, $CD19^-$, $CD3^+$ and $CD4^+$/ $CD8^+$ T cells. To determine T cell response, $CD4^+$ and $CD8^+$ T cells were gated on IFN-γ and TNF.
